# Diphenoxylate Toxicity in a Young Child with Acute Gastroenteritis: A Clinical Case Report

**DOI:** 10.15190/d.2024.20

**Published:** 2024-12-31

**Authors:** Muhammad Romail Manan, Iqra Nawaz, Ahmad Zulaid, Muhammad Usama Afzal, Muhammad Atif, Muhammad Huzaifa, Anosha Fayyaz

**Affiliations:** ^1^Faculty of Medicine, Services Hospital, Lahore, Pakistan; ^2^Faculty of Medicine, Bahawal Victoria Hospital, Bahawalpur, Pakistan; ^3^Faculty of Medicine, Quaid-e-Azam Medical College, Bahawalpur, Pakistan

**Keywords:** Diphenoxylate, atropine, poisoning, drug-related side effects, drug-related adverse reactions.

## Abstract

Lomotil (diphenoxylate-atropine) toxicity in the pediatric population remains a significant concern particularly in low and lower middle-income countries. This may result from accidental ingestion or inappropriate therapeutic administration which can lead to life threatening complications including respiratory and central nervous system depression. A 2-year-old child presented to the pediatric emergency room in an altered state of consciousness. Clinical examination revealed dry mucous membranes, and a prolonged capillary refill time with weak radial pulses. Keeping in view the one-day history of 10-12 episodes of acute onset loose, watery stools, patient was initially treated as a case of hypovolemic shock. With rehydration therapy, his perfusion improved. However, the Glasgow Coma Scale score remained 8, as was observed on initial presentation. Upon further probing, it was revealed by the parents that the child had been given Lomotil by a local general practitioner for unresolved watery diarrhea. Pinpoint pupils and slow shallow vesicular breathing confirmed this diagnosis of Lomotil overdose. Administration of 0.1mg/kg/dose Naloxone repeated once, completely reversed the toxic effects. The child was able to make a full recovery and was discharged the following day. This case highlights the importance of recognizing and managing diphenoxylate toxicity in children, emphasizing the need for increased clinical awareness. A lack of consensus regarding the toxic dose of this drug reveals a gap warranting further research and establishment of standardized guidelines to ensure accurate dosing and improved patient safety.

## INTRODUCTION

Among other causes of mortality described in children under five years of age, diarrhea continues to be the second leading cause of death^1^. Infantile diarrhea, which can occur from birth to 24 months ofage, warrants immediate medical attention^2^. Although the first line management of infantile diarrhea involves rehydration through oral or intravenous route, pharmacotherapy with antidiarrheals may be used as an adjuvant^3^.

Diphenoxylate is an active ingredient in commonly prescribed antimotility drugs^4^. This opioid receptor agonist is only available in combination form with a subtherapeutic dose of atropine added in order to reduce the potential of abuse and overdose^3^. This combination is also referred to as Lomotil. One tablet or 5cc syrup of Lomotil is composed of 0.025 mg of atropine sulfate, an anticholinergic, and 2.5 mg of diphenoxylate^3^.In 24 hours, a maximum of eight tablets (20 mg of diphenoxylate) can be administered in an adult^5^. Insufficient evidence exists to establish a safe dose of this drug in children under the age of 13 years. However, it should be noted that this combination is not recommended for children under six years of age due to the potential for severe complications^5^. Diphenoxylate poisoning resulting from improper dosing is relatively frequent, especially among children aged below 10 years ^1^. Immature liver function further contributes to a slow drug metabolism and prolonged stay in the body which then contributes to toxic effects^4^.

Administration of doses exceeding 20 mg is linked to symptoms indicative of toxicity. Depression of the central nervous system (CNS) is a well-documented adverse effect particularly in children under the age of six years. It is important to note that as the blood brain barrier (BBB) is imperfect in children, this drug can suppress the CNS by crossing BBB and exerting an inhibitory effect^4^. Other CNS effects include lethargy, malaise, drowsiness, sedation, numbness of extremities, restlessness, headache, confusion, dizziness, and hallucination. Lomotil at toxic doses also leads to respiratory failure in children below six years. Diphenoxylate-induced hypoxia was reported as the major complication in a review of 28 cases by McCarron and colleagues^6^. Anticholinergic adverse effects include tachycardia, increased body temperature, retention of urine, and dry mucous membranes and skin. This combination (diphenoxylate-atropine) can also lead to gastrointestinal complications resulting from increased transit time and decreased gut motility that contribute to overgrowth of bacteria and enterotoxins release^5^.

In this case report, diphenoxylate-atropine toxicity is described in a 2-year-old child who was prescribed this drug for the management of acute diarrhea.

## CASE REPORT

A 2-year-old child presented to the Pediatric Emergency Room of Services Hospital, Lahore with disturbed consciousness. He had a one-day history of 10-12 episodes of acute onset loose, watery stools which were profuse, foul-smelling, easy to wash, and did not contain gross blood or mucus. According to the parents, he was completely vaccinated as per the current Expanded Program on Immunization (EPI) schedule and had no notable past history. His initial assessment has been summarized below:

- Vital signs: Heart rate (122bpm), Respiratory rate (12/minute), Temperature (98.8 F), Oxygen saturation (92%)

- Glasgow Coma Scale (GCS): 8/15 – E2M4V2

- General Physical Examination: Dry mucous membranes, prolonged capillary refill time, weak radial pulses, pinpoint pupils

Gastrointestinal examination revealed a flat abdomen with midline umbilicus and no palpable visceromegaly. Furthermore, he was unconscious and unable to drink, hence he was classified presumptively as being in hypovolemic shock. Rehydration therapy with 20ml/kg bolus of Normal Saline was commenced immediately. On reassessment after 15 minutes, no improvement was observed in the GCS of the patient, however, pulses and capillary refill time had normalized. Full blood count, serum electrolytes, and arterial blood gases were ordered (Table 1).

Further it was revealed by the parents that the child could not be roused since morning after being given Lomotil by a local general practitioner. Parents were unaware of the quantity of medication used. This correlated with the decreased respiratory rate and pinpoint pupils observed previously upon initial assessment. At this point, diphenoxylate-atropine toxicity was established as the diagnosis, and treatment using Naloxone was initiated immediately. The effect was completely reversed after therapy with two doses of 0.1mg/kg/dose Naloxone given at an interval of one minute. Rehydration therapy was initiated according to the Integrated Management of Newborn and Childhood Illnesses (IMNCI) in the emergency room, and later, the child was admitted to pediatric intensive care unit (PICU) for further monitoring. He recovered completely and was discharged with advice regarding Oral Rehydration Therapy (ORT) adjunctive with probiotics and zinc supplements.

**Table 1:** Complete blood picture of the patient

Full Blood Count	Observed Value	Normal Value
White cell count (10^9^/L)	10.9	4-11
Red cell count (10^12^/L)	4.5	4-5.5
Hemoglobin (g/dL)	11.2	11-16
Hematocrit (%)	48.2	40-52
Mean corpuscular volume (fl)	92.3	82-95
Mean corpuscular hemoglobin (pg)	31.9	27-31
Mean corpuscular hemoglobin concentration (g/dL)	35.6	32-36
Neutrophil (%)	34.2	40-70
Lymphocyte (%)	46.2	20-45
Platelet count (10^3^/μl)	390	150-450

## DISCUSSION

The major therapeutic component of the combination, diphenoxylate, is a derivative of phenylpiperidine, related to meperidine^7^. It acts on the on μ-opioid receptors located in the enteric nervous system to inhibit acetylcholine being released, leading to a decrease in the gastrointestinal contractions which results in prolongation of gastric emptying and ultimately in an increase in the transit time of intestinal contents^7^. Additionally, it exerts antisecretory effects and increases active absorption via its action on the delta receptors^5^. Diphenoxylate has been categorized as a schedule II drug under Food and Drug Administration^5^. Schedule II drugs have a high potential for abuse with high psychological and physical dependence. This tendency of abuse is countered by the addition of atropine sulfate which makes it a schedule V drug ^5^. Though the dose of atropine sulfate is small for adults, children can display symptoms of atropinism even when administered standard doses^8^.

The efficacy and safety of the use of Lomotil among the pediatric population remains a matter of significant concern. Although there appears to be a lack of credible evidence establishing safety of Lomotil in children below the age of 13 years, several case reports have documented the toxic effects resulting from its poisoning following inappropriate dose administration or accidental ingestion.

Diphenoxylate is metabolized to diphenoxylic acid (difenoxin) which is a biologically active metabolite and exhibits greater potency and a longer duration of action compared to diphenoxylate^1^. Given the slow absorption and prolonged excretion, the symptoms of Lomotil poisoning may be delayed or can recur^8^. As the absorption is delayed due to a decrease in gastric motility, the adverse effects are further exacerbated leading to secondary poisoning^9^. Furthermore, the drug metabolism may be slow in children, given the immature liver function^4^. The slow absorption and prolonged duration of action was described in a child who developed apnea for 10 hours after ingestion of Lomotil tablets followed by a second episode of apnea 7 hours later^10^. Curtis and Goel, while evaluating 45 cases of diphenoxylate-atropine toxicity in children, established that there was no relationship between the amount of Lomotil ingested and the intensity of the subsequent symptoms^8^. They reported that in the group with mild symptoms, the average dose of diphenoxylate and atropine was higher (29 and 0.029 mg/kg) compared to those with moderate symptoms (23 and 0.023 mg/kg)^8^. Thus, it becomes challenging to ascertain the dose which may cause toxicity in children.

Lomotil toxicity typically occurs in two phases. The first phase, which is mild, can be attributed to the anticholinergic effect of atropine, resulting in tachycardia, drowsiness, restlessness, and constipation. These features persist for about three hours after which the symptoms of diphenoxylate appear to dominate. However, it has been reported that Lomotil intoxication is primarily characterized by symptoms of opioid overdose and less commonly due to atropine toxicity. Features of opioid intoxication dominated and were observed without any effects of atropine excess in 92% of the cases^6^. This second phase is largely due to the effects rendered by diphenoxylate resulting in respiratory depression and CNS suppression. Wang et al. have reported anoxic-ischemic encephalopathy manifesting as multiple complex partial seizures following Lomotil overdose in a 3-year-old child^4^. Additionally, respiratory depression is one of the most fatal side effects of Lomotil, requiring immediate resuscitation^5^, as highlighted by Ghosh et al., who reported a case of a 1.5-month-old with Lomotil overdose requiring admission to the pediatric intensive care unit^1^. This underscores the need for healthcare professionals to remain vigilant about the toxic effects of such substances, given the gravity of their potential consequences.

Lomotil intoxication can be managed with early and appropriate intravenous administration of naloxone at 0.05–0.1 mg/kg among other modalities of treatment available including the use of emetics, performing gastric lavage, and activated charcoal, forced diuresis^3^.

## CONCLUSION

Diphenoxylate toxicity in the pediatric population remains a significant concern particularly in low- and middle-income countries (LMICs). Healthcare professionals globally, especially those in LMICs, need to maintain caution while prescribing this combination in order to prevent life-threatening complications. This case serves as a strong reminder that risks associated with overdosing of Lomotil are not widely acknowledged. Furthermore, the lack of research establishing the effectiveness and safety of this drug in pediatric patients indicates a lack of attention towards this issue. As a result, healthcare professionals may remain uninformed about the potential doses at which this drug can cause toxicity. Thus, through this case report we urge the need for further research in order to establish safe dosing strategies for pediatric patients, emphasize the need for updated protocols and clearer recommendations and stress on the importance of initiating and continuing education and awareness programs to prevent poisoning incidents.

## Major points

- Lomotil (diphenoxylate-atropine) overdose can result in life threatening complications including significant respiratory and central nervous system suppression, particularly in the pediatric population.

- Lack of research on the exact dose of Lomotil causing toxicity in children coupled with the absence of evidence-based data contributes to the inappropriate prescription of this drug

- This case report serves as a call to action by highlighting the toxic effects of this drug and by emphasizing the urgent need for developing clear dosing guidelines, and stronger regulatory measures
